# Skin Fragility-Woolly Hair Syndrome (SFWHS): A Case Report

**DOI:** 10.7759/cureus.100931

**Published:** 2026-01-06

**Authors:** Ahmed Almousa, Saud Binsufayan, Mohammad K Almazied, Tasneem Aldraye, Saud Alsharif

**Affiliations:** 1 Dermatology, Security Forces Hospital, Riyadh, SAU; 2 Pediatrics, Security Forces Hospital, Riyadh, SAU; 3 Dermatology, Imam Mohammad Ibn Saud Islamic University, Riyadh, SAU

**Keywords:** dsp mutation, nail abnormalities, palmoplantar keratoderma, skin fragility-woolly hair syndrome (sfwhs), woolly hair

## Abstract

Skin fragility, palmoplantar keratoderma, and characteristic woolly hair are defining features of skin fragility-woolly hair syndrome (SFWHS), an extremely rare inherited disorder. This condition is classified within the group of desmosomal disorders, which are associated with compromised structural integrity of the skin and hair. Clinically, affected individuals often present with hair fragility, fragile skin, recurrent erosions, nail abnormalities, and varying degrees of palmoplantar thickening beginning in early childhood. In this case report, we describe a patient with this extremely rare syndrome.

## Introduction

Skin fragility, palmoplantar keratoderma, and characteristic woolly hair are the hallmarks of the extremely rare inherited disorder skin fragility-woolly hair syndrome (SFWHS). This condition belongs to the group of desmosomal disorders, which are characterized by disruptions in the integrity of the skin and hair. The DSP (desmoplakin) gene, which encodes desmoplakin, is a key component of desmosomes, anchoring intermediate filaments and maintaining intercellular cohesion, and is among the most frequently implicated genes. Mutations in DSP, particularly those inherited in an autosomal recessive manner, compromise this structural framework, leading to the distinctive phenotype [1-3].

In dermatology, DSP mutations are mainly associated with two syndromes: Carvajal syndrome, which involves mutations in the C-terminal tail of DSP and affects both the skin and heart, and SFWHS, first described in 2011, caused by mutations in the N-terminal head of DSP. SFWHS is similar to Carvajal syndrome but is limited to cutaneous manifestations [1]. Herein, we report a case of SFWHS.

Recent advances in genomic technologies have enabled early recognition of DSP mutations, including those leading to cutaneous-limited and cardio-cutaneous syndromes. Early identification of DSP mutations allows physicians to anticipate extracutaneous involvement, arrange appropriate follow-up, and implement early interventions. Furthermore, genetic counseling can help prevent these syndromes in families carrying recessive DSP mutations [1-3].

## Case presentation

**Figure 1:**
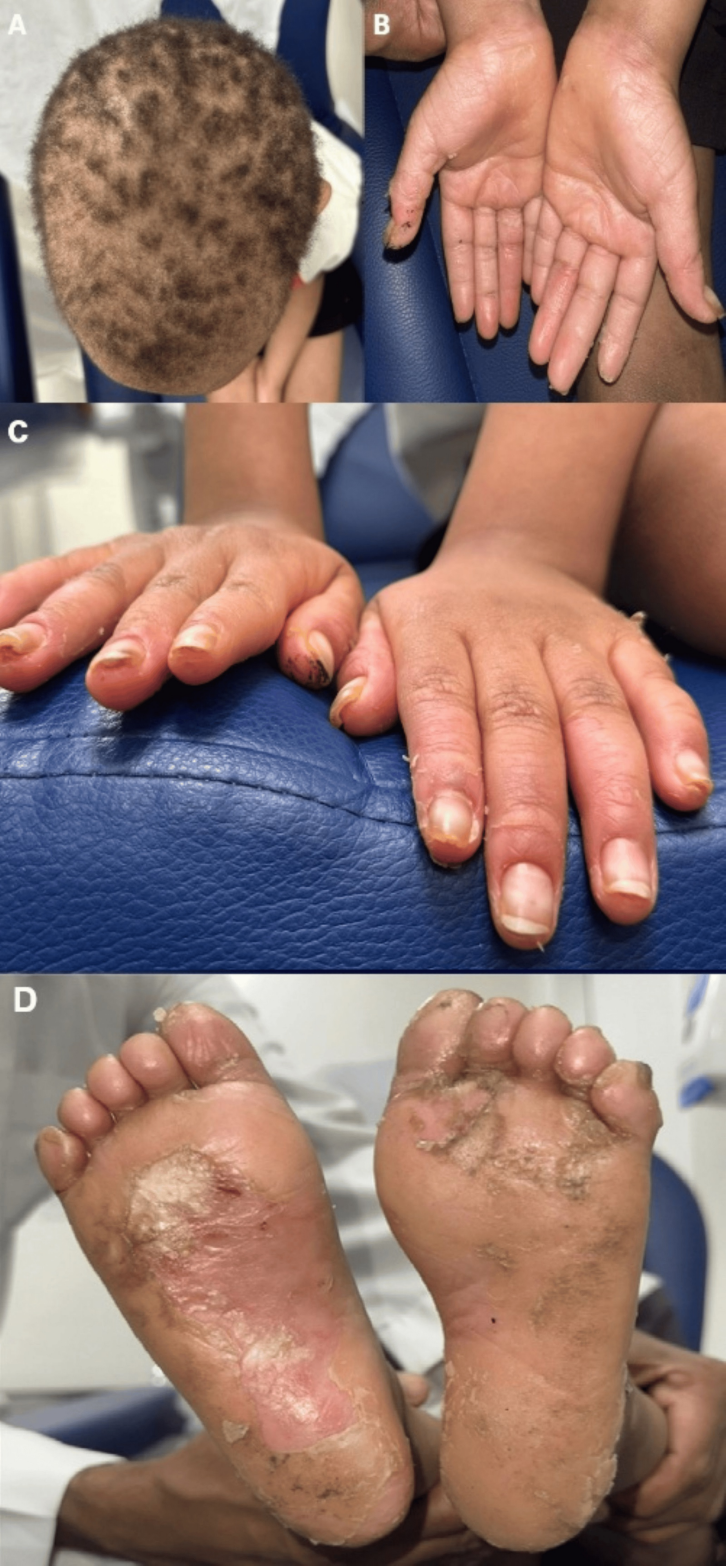
Clinical features of SFWHS Coarse and woolly hair (A), multiple palmoplantar keratoderma over both palms (B), subungual hyperkeratosis (C), and multiple plantar keratoderma with eroded skin over both soles (D). SFWHS, skin fragility-woolly hair syndrome

A six-year-old boy, born full-term via normal vaginal delivery with no history of neonatal intensive care unit admission or developmental delay, presented to the clinic with a history of recurrent erosions and blisters involving the palms and soles since the age of one year. He was born to consanguineous parents who are medically healthy with no similar symptoms or medical conditions, and he has one older brother, aged 10 years, who is also medically well and does not exhibit similar symptoms. On physical examination, diffuse palmoplantar keratoderma was observed over the palms and soles, accompanied by woolly hair, subungual hyperkeratosis, and onychogryphosis, as shown in Figure 1.

**Figure 2:**
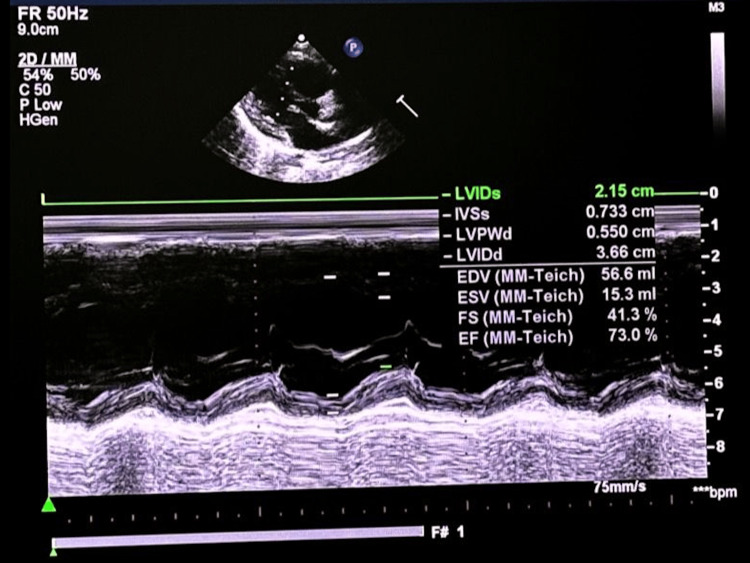
Echocardiogram showing normal cardiac anatomy with an ejection fraction of 73%

There was no history of cardiac symptoms at presentation. The patient was referred to genomic medicine, where whole-exome sequencing revealed a homozygous likely pathogenic variant in the DSP gene, consistent with an autosomal recessive diagnosis of SFWHS. After confirming the diagnosis, the family was counseled and educated about the syndrome. The patient was subsequently referred to cardiology, where echocardiography demonstrated normal cardiac anatomy and a preserved ejection fraction of 73% (Figure 2).

From a dermatological perspective, management involves educating the family to prevent mechanical trauma, applying topical 20% urea over areas of keratoderma, using emollients to enhance skin health, and applying topical antibiotics to eroded skin or wounds. The family also received genetic counseling and education regarding the condition.

## Discussion

DSP mutations in dermatology are primarily associated with Carvajal syndrome, a condition characterized by both cutaneous and cardiac manifestations. SFWHS was first described in 2011. The main difference between Carvajal syndrome and SFWHS lies in the location of the mutation within the DSP gene. Carvajal syndrome typically affects the C-terminal domain of desmoplakin, which binds intermediate filaments. Disruption of this domain impairs the mechanical connection between desmosomes and the cytoskeleton, accounting for the significant cardiac involvement, particularly dilated or arrhythmogenic cardiomyopathy, as well as woolly hair and palmoplantar keratoderma [1-3].

In contrast, SFWHS involves mutations in the N-terminal head of desmoplakin, sparing the C-terminal tail. This results in predominantly cutaneous manifestations without consistent cardiac involvement. Preservation of partial desmoplakin function may explain the absence of cardiac disease in SFWHS while still producing significant epidermal and hair shaft instability. Thus, the location of the mutation is an important predictive factor for cardiac involvement [1].

SFWHS remains extremely rare, with few reported cases in the literature, as summarized in Table 1 [1].

**Table 1:** Reported cases of SFWHS SFWHS, skin fragility-woolly hair syndrome

Study	Number of patients	Cutaneous features	Hair findings	Nail involvement	Cardiac involvement
Al-Owain et al. (2011) [1]	5	Skin fragility with recurrent erosions and palmoplantar keratoderma	Woolly hair	Variable nail involvement	No cardiac involvement
Peter et al. (2018) [4]	1	Skin fragility and palmoplantar keratoderma	Woolly hair	Variable nail changes	No cardiac involvement
Yang et al. (2025) [5]	1	Skin fragility affecting trauma-prone areas and palmoplantar keratoderma	Woolly/curly hair	Marked nail dystrophy/pachyonychia	No cardiac involvement

Herein, we report a case of SFWHS in which the patient demonstrated hallmark features of the syndrome, including palmoplantar keratoderma, hair fragility, and nail changes, with no cardiac involvement, confirmed by genetic testing.

In 2018, Peter et al. reported a case of a 23-year-old female with woolly hair and palmoplantar keratoderma since early childhood, with no cardiac involvement; genetic testing confirmed SFWHS [4]. In 2025, Yang et al. reported a two-year-old Chinese boy with palmoplantar keratoderma and woolly hair. There were no cardiac symptoms, and echocardiography was normal [5].

Importantly, SFWHS is not known to cause cardiac abnormalities. However, other DSP mutations can lead to arrhythmogenic cardiomyopathy, so periodic cardiac screening is recommended [1].

Management of SFWHS is primarily supportive, focusing on symptom control, prevention of secondary complications, and improvement of daily functioning. Treatment options range from topical emollients and keratolytics to systemic retinoids such as acitretin. Genetic counseling is essential, particularly in populations with high rates of consanguinity who present with these clinical features [1,2].

SFWHS is considered a dermatologic disorder without extracutaneous involvement, whereas other DSP variants may have extracutaneous manifestations, mainly cardiac. Reported DSP mutations exhibit variable phenotypes, ranging from purely cutaneous involvement to cardiocutaneous syndromes. This diversity underscores the importance of serial cardiac screening, even in the absence of initial symptoms, as cardiac manifestations may develop later. Early detection and supportive treatment, including therapy targeted to palmoplantar keratoderma, wound care, and psychosocial support, may improve outcomes. Ongoing reports of rare cases, such as the present report, provide practical insights into the evolving clinical approach to recessive DSP-associated disorders [1-3].

## Conclusions

SFWHS is a rare hereditary disorder that can be challenging to diagnose and manage. This case report highlights the importance of early recognition and comprehensive evaluation, including genetic testing and cardiac assessment, to improve quality of life through timely skin-supportive management and, importantly, to rule out cardiac involvement and reduce potential morbidity. Dermatologists play a key role in early detection, long-term management of cutaneous complications, and counseling families regarding the genetic implications of the disorder.
